# Native freshwater species get out of the way: Prussian carp (*Carassius gibelio*) impacts both fish and benthic invertebrate communities in North America

**DOI:** 10.1098/rsos.170400

**Published:** 2017-10-04

**Authors:** Jonathan L. W. Ruppert, Cassandra Docherty, Kenton Neufeld, Kyle Hamilton, Laura MacPherson, Mark S. Poesch

**Affiliations:** 1 Department of Renewable Resources, University of Alberta, 751 General Services Building, Edmonton, Alberta, Canada T6G 2H1; 2 Alberta Environment and Parks, Fish and Wildlife Division, 6909-116 Street, Edmonton, Alberta, Canada T6H 4P2

**Keywords:** before–after comparison, time since invasion, freshwater fishes, benthic invertebrates, non-native species, concordance

## Abstract

Prussian carp (*Carassius gibelio*) are one of the most noxious non-native species in Eurasia. Recently, Prussian carp, a non-native freshwater fish species, were genetically confirmed in Alberta, Canada and have been rapidly expanding their range in North America since establishment. Given their rapid range expansion, there is an increasing need to determine how Prussian carp may impact native species. We assessed the severity of the Prussian carp invasion by (i) determining their impact on fish communities, (ii) assessing their impact on benthic invertebrate communities, (iii) evaluating if Prussian carp alter abiotic conditions, and (iv) identifying where we find higher abundances of Prussian carp. When Prussian carp were established, we found significant changes to the fish community. Correspondingly, the degree of impact to benthic invertebrate communities was related to the stage of invasion (none, early or recent), where changes in fish communities were significantly concordant with changes in benthic invertebrate communities. Finally, we found that higher abundances of Prussian carp were significantly associated with lower abundances of a majority of native fish species. Altogether, using three lines of evidence, we determine that Prussian carp can have wide-ranging impacts on freshwater ecosystems in North America, pressing the need for management intervention.

## 1. Introduction

Globally, biotic invasions are recognized to be one of the largest extinction threats to species [1–3]. This not only poses major issues with maintaining native biodiversity, but also the resilience and function of the native ecosystems when non-natives establish [4,5]. The estimated number of non-native species introduced to the USA, South Africa, Australia, India, UK and Brazil exceeds 120 000 species across all taxa [6]. This, in turn, is estimated to have a combined cost of $314 billion USD annually due to damages [6]. For aquatic species in freshwater ecosystems, the impact of non-native species is disproportionately higher [1,7]. This is a consequence of the natural constraints of freshwater biogeography [7]. Isolation and dispersal limitation in freshwater species result from the arrangement of dendritic networks or physiological barriers at fine spatial scales and there is also an inability for freshwater species to traverse expansive oceanic, desert or mountain environments at broader spatial scales [7]. This low connectivity of aquatic organisms in freshwater ecosystems promotes endemism and speciation, where novel opportunities to disperse have recently come about via human-mediated dispersal, which has subsequently increased the invasion probability for many aquatic organisms [8,9].

In North America, freshwater ecosystems are imperilled by many non-native fish species. One notable example has been the establishment, spread and impact of Asian carp (the colloquial term for silver carp (*Hypophthalmichthys molitrix*), bighead carp (*H. nobilis*), grass carp (*Ctenopharyngodon idella*) and black carp (*Mylopharyngodon piceus*)), which now threaten the function of many freshwater ecosystems [10,11]. Since their introduction to North America in the 1970s, their range has expanded throughout most of the Mississippi river drainage [11] and more recently within the Great Lakes region [12]. In terms of impact to native biodiversity and ecosystems, Asian carp have been shown to influence the trophic structure of fish communities and the abiotic factors that support native freshwater fish communities [10].

Recently, Prussian carp (*Carassius gibelio*), one of the most harmful non-native species found in Eurasia, were confirmed in western North America [13,14]. The origin of Prussian carp has been identified in some parts of Europe (from the Baltic Sea to the Mediterranean) [15] and northern China [16]. While not all invasive species have negative impacts on native species (e.g. [17]), Prussian carp has a high trait overlap (e.g. feeding, habitat preferences) with many Asian carp species that do impact native North American fish species and ecosystems [10,11,18]. Since their establishment in North America in 2000, Prussian carp have shown a high rate of spread, doubling their spatial extent approximately every 5 years. Further, the distance of current Prussian carp occurrences is within 200 km of the Missouri drainage, where they have the potential to spread across much of the continental USA [18]. As Prussian carp are morphologically similar to goldfish (*C. auratus*) and common carp (*Cyprinus carpio*), there is a high likelihood of misidentification and delayed detection in newly invaded systems; which is probably the case in western North America [13,15]. The potential range expansion and impact of Prussian carp on native species is a concern for conservation managers.

Potential concerns associated with Prussian carp in North America are numerous. In Eurasia, Prussian carp are known to aggressively colonize new habitats and become the most dominant species, which ultimately outcompetes most native species [19]. They possess a number of qualities that make them well suited to establish and spread, including: the ability to tolerate extreme environmental conditions such as low oxygen, eutrophication and high turbidity while consuming an omnivorous diet consisting of amphibians, molluscs, annelids, macrophytes, detritus and invertebrates [20,21]. Uniquely for a fish species, they are also capable of reproducing asexually through a process referred to as gynogenesis [22]. This reproductive process parasitizes sperm from other Cyprinidae fish species to initiate the development of eggs [13,23,24]. Altogether, these life-history characteristics are a major concern for displacement of native species, because Prussian carp are a generalist species that can survive in extreme environmental conditions, while reproducing via gynogenesis which ‘interferes’ with native species spawning [18]. Further, Prussian carp have been shown to degrade and alter habitat quality by disturbing sediment during foraging, furthering declines in native fish species [14,20,25]. Thus, Prussian carp have numerous life-history traits that make it a potential noxious invader that can alter fundamental ecological processes in freshwater ecosystems in North America.

Using a combination of historical survey records and field sampling, we address knowledge gaps related to the potential risk of Prussian carp by assessing if their establishment impacts: (i) fish community structure, (ii) benthic invertebrate community composition, and (iii) abiotic factors within the ecosystem. We also determined what factors may be related to increases or decreases in Prussian carp abundance across their North American range. The findings from this study will better inform our understanding of the potential impact of Prussian carp in North America. Moreover, it will provide insight into whether it is possible to intervene with their continued range expansion and reduce impacts on native species.

## 2. Material and methods

### 2.1. Study area

**Figure 1.**
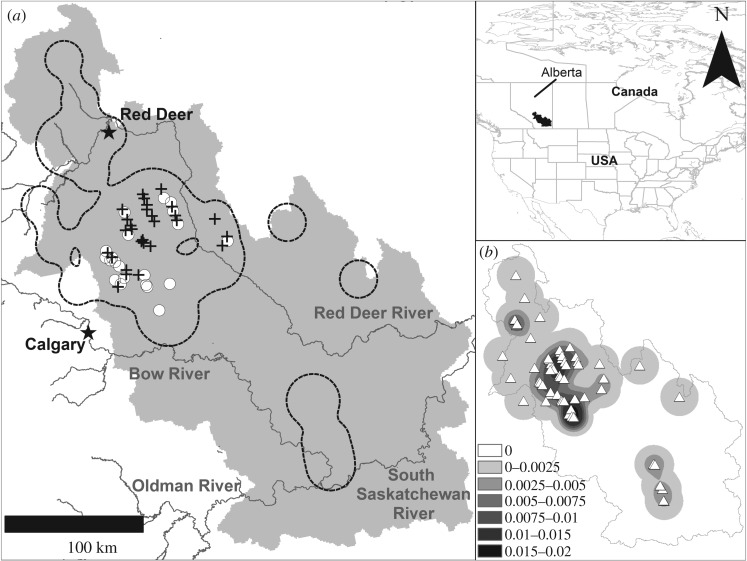
(*a*) The 2014 sampling locations in the Red Deer River watershed (light grey) located within Alberta, Canada. Sites with only samples from 2014 are denoted by white circles and those sites with samples from 2005 to 2014 are denoted by a cross. The dashed black line is the estimated range extent of Prussian carp as of 2014. (*b*) All confirmed presences of Prussian carp (white triangles) as of 2014 overlaid on the kernel density estimate (number of presences per 0.25 km^2^).

We conducted field surveys in 2014 on 12 streams within the Red Deer River watershed (51°40.407′ N, 113°18.707′ W), approximately 130 km northeast of Calgary, Alberta, Canada (figure 1). This area is situated at the intersection of four different ecoregions: Northern Fescue, Foothill Fescue, Central Parkland and Mixed Grassland [26]. The dominant anthropogenic activity is agriculture, which can occupy over 85% of the landscape in some areas, followed by urbanization and industrial activities [26,27]. Site selection for sampling was based on areas where Prussian carp was not yet known to be established as of 2005 [28], but thought to occur as of 2014 (T. Clayton 2016, personal communication). Field surveys in 2014 occurred across 42 sites in the Red Deer River watershed, including the following tributaries: Ghostpine Creek, Three Hills Creek, Kneehills Creek, Lonepine Creek, Rosebud River, Carstairs Creek, Crossfield Creek, West Michichi Creek, Michichi Creek and three unnamed streams (figure 1*a*). Sampling of sites in 2014 was concordant with 28 sites that were sampled in 2005 (figure 1*a*).

### 2.2. Field sampling

Each sampling site consisted of a 300 m wadeable stream or river section, and was sampled for the fish community, flow, depth, water quality (pH, dissolved oxygen, temperature, turbidity, electrical conductivity, phosphate, nitrate), fine substrate (per cent silt and clay) and amount of aquatic vegetation [29]. Electrofishing was conducted using a standardized single-pass electrofishing procedure in an upstream direction with a Smith-Root LR-24 backpack electrofisher for an average of 1734 s (502–3173 s) [29]. The targeted survey time was 1500 s to ensure adequate sampling of the fish community [30]. However, variations in sampling effort were due to differences in site characteristics (e.g. stream width, depth).

At each sample site, all species caught were identified and enumerated. All species were standardized to catch per unit effort (CPUE) as the abundance per second per metre square of area surveyed (hereafter referred to as abundance). This allowed us to reduce the data bias related to different amounts of effort for both the time and area sampled [31]. After fish collection, a subset of sites (*n* = 22) were sampled for benthic invertebrates and all sites were sampled for habitat. Benthic invertebrates were sampled using standardized kick-net samples, consisting of a 2 min sample of a 2 m^2^ area with a 253 µm mesh net within the site [32]. After collection, benthic invertebrates were frozen and stored at −20°C until identification. Identification of benthic invertebrates to family level at each site was completed in the laboratory [33] (see electronic supplementary material, table S1 for list of families). Habitat data, stream width and water depth (mean and maximum) were measured at three points across sites and then averaged for each site. Water quality parameters were measured midway across the stream in a location representative of the site. Dissolved oxygen (±0.2 mg l^−1^), conductivity (± 0.01 µS cm^−1^) and pH were recorded using a multimeter (YSI, Yellow Springs, Ohio). Turbidity samples were collected using a portable turbidity meter (LaMotte 2020we; ±50 nephelometric turbidity units, NTU) in the upper 30 cm of the water column and stream velocity was measured at mid-water depth. Water samples were analysed for soluble phosphate and nitrogen using the colorimetry method by the Natural Resources Analytical Laboratory at the University of Alberta [34,35]. Finally, per cent cover of aquatic vegetation and stream substrate were estimated through visual assessment of the entire 300 m sampling site [29].

### 2.3. Impacts of Prussian carp on native biota

Fish and habitat data were collected within the Red Deer River watershed in 2005, prior to the invasion of Prussian carp [28]. Standard protocols for fish sampling and habitat data collection were used (see above for details); however, the total shock time was on average 373 s (ranging 206–554 s) and only 60–80 m stream segments were sampled. Thus, there is a difference in sampling effort between 2005 and 2014, because the time and area sampled is not similar. Hence, all species abundances were standardized as the abundance per second per metre square of area surveyed. Out of the 42 sites sampled in 2014, 28 of the sites where Prussian carp were present were also sampled in 2005, meaning that a total of 28 sites could be compared before and after Prussian carp invasion.

The impact of Prussian carp establishment was also assessed by estimating the time since invasion by calculating the potential range extent of Prussian carp over the time frame of 2000–2014 using confirmed occurrences from Elgin *et al*. [13], Alberta Environment and Parks [36], and specimens collected in this study (figure 1*b*). To estimate the range extent for each year, we performed a kernel density using confirmed presence records and a 25 km bandwidth in *ESRI ArcGIS 10.2*, following methodology established by Worton [37] (please see [18] for data and details). Briefly, using the presences of Prussian carp, kernel density provides a spatial estimate of the density of presences and allows one to interpret areas where there are more or less presences across their detected range (i.e. provides areas of greater certainty of a presence or absence; see electronic supplementary material, figure S1). We then used the kernel density surfaces to estimate the spatial contour of the 95th percentile of the density surface, which is the estimated range extent of Prussian carp. This was conducted in *ESRI ArcGIS 10.2* and *Geospatial Modeling Environment* and it will reduce the influence of geographically extreme presences, which can drastically influence this range extent calculation [38]. Each sample location where Prussian carp were found in the 2014 sampling was then attributed an estimated time that Prussian carp could have invaded the site over the 2000–2014 period. We then classified sites where Prussian carp were established based on the estimated time of invasion as early (4–9 years) or recent (1–3 years), which are representative of many generations (greater than 2) and only a couple of generations (less than or equal to 2) of establishment, respectively (assuming spawning three times per spawning season per year and that the age of maturity is half a year old) [15,18].

We assessed differences in community composition using principal components analysis (PCA) and Hellenger transformed abundances of species [39,40]. To do so, we used the *decostand* and *cca* function in the *vegan* library for the R software [41]. Prior to analysis, we ensured that species were detected in more than 5% of sites, in order to remove any effects of rare species on the PCA analysis [42]. All fish species (*n *= 7) were detected in greater than 5% of sites sampled. First, using PCA we investigated fish community differences at sites (*n *= 28) before and after Prussian carp were established. Secondly, we assessed differences in fish communities by the time since invasion: no invasion (*n* = 13), early invasion (*n* = 11) and recent invasion (*n* = 18; see above for details). Thirdly, differences in benthic invertebrate communities were investigated at sites that had no Prussian carp (*n *= 8), early Prussian carp invasion (*n *= 5) or recent Prussian carp invasion (*n *= 9) using PCA (electronic supplementary material, table S1). To test for differences in the fish and benthic invertebrate communities at sites, we compared before–after and time since establishment of communities using permuted multivariate analysis of variance (PERMANOVA) [43]. Moreover, we ran the PERMANOVA with and without Prussian carp abundance included to determine if community compositional impacts by Prussian carp were independent of changes in Prussian carp abundance. This was conducted using the *adonis* function in the *vegan* package for the R software [41]. We also tested for differences in the abundance of each native species using permuted *t*-tests (before–after) and one-way analysis of variance (ANOVA; time since invasion) to determine if we could detect any direct impacts to native species abundance [44,45]. Finally, we tested for concordance between fish and benthic invertebrate communities, for sites classified by the time since invasion, using a Procrustes rotation analysis. This is achieved by testing for the degree of similarity between two ordination solutions (i.e. the PCAs), where we used 10 000 permutations to test for significance [46]. Here, we used the *protest* function in the *vegan* package in the R software [41].

### 2.4. Impact of Prussian carp on habitat and abiotic factors

To evaluate differences in habitat and the overall native species abundance in sites sampled in 2005 (before) and 2014 (after; *n *= 28), we used permuted *t*-tests with 10 000 permutations [44]. Habitat variables collected during both periods included: dissolved oxygen, electrical conductivity, fine substrate (silt and clay), turbidity, mean depth and flow rate. We also compared differences at sampling locations from 2014 that had no Prussian carp, early Prussian carp invasion or recent Prussian carp invasion, using a one-way permuted ANOVA [45]. This included all habitat variables collected (see above for details). Permuted *t*-tests and ANOVAs were desirable given that they are suitable for unbalanced and non-parametric comparisons [47]. Finally, all *p*-values were adjusted for multiple comparisons using the Bonferroni method. Analysis was conducted in the R software for statistical computing [48].

### 2.5. Environmental predictors of Prussian carp abundance

To determine what factors promoted or decreased the abundance of Prussian carp, we implemented a generalized linear mixed model (GLMM) to assess the magnitude and direction of impact of both abiotic and biotic variables [49]. The random effect included in the model was time since invasion as sites were classified as no Prussian carp, early Prussian carp invasion or recent Prussian carp invasion. To maximize the sample size (*n* = 40), phosphate (PO_4_; mg l^−1^) and nitrate (NO_3_; mg l^−1^) were dropped as co-variables for this analysis. Co-variables included in the GLMM analysis were: dissolved oxygen (mg l^−1^), electrical conductivity (µS cm^−1^), fine substrate (% sand and clay), turbidity (NTU), aquatic vegetation (%), flow rate (m s^−1^), pH, mean depth (m) and the abundances of each native fish species. Prior to analysis, all variables were assessed for collinearity using Pearson correlation (pairwise *r *≥ 0.8 were assessed as collinear) and abiotic variables were standardized to *z*-scores and centred, as variables were on different scales and units of measurement. Further, to be consistent with previous analysis, we used Hellenger transformed abundances. Backward elimination was conducted on variables selected in the final model and was performed using the *lmerTest* package in the R software [50]. Model fit of the full and reduced models was conducted by evaluating the corrected Akaike information criterion (AICc) and log-likelihood (LL). Finally, residuals were evaluated for goodness of fit (results not shown). This analysis was conducted using the *lmer* function in the *lme4* package in the R software [51].

## 3 Results

Sites sampled in this study included those with no Prussian carp invasion (*n *= 13), early Prussian carp invasion (*n *= 11; 4–9 years ago) and recent stage of invasion (*n *= 18; 1–3 years ago). Altogether, native species captured in this study included: fathead minnow (*Pimephales promelas*), white sucker (*Ca. commersoni*), lake chub (*Couesius plumbeus*), brook stickleback (*Culaea inconstans*), longnose dace (*Rhinichthys cataractae*) and longnose sucker (*Ca. catostomus*).

### 3.1. Before–after and time since invasion impacts on native fishes

**Figure 2.**
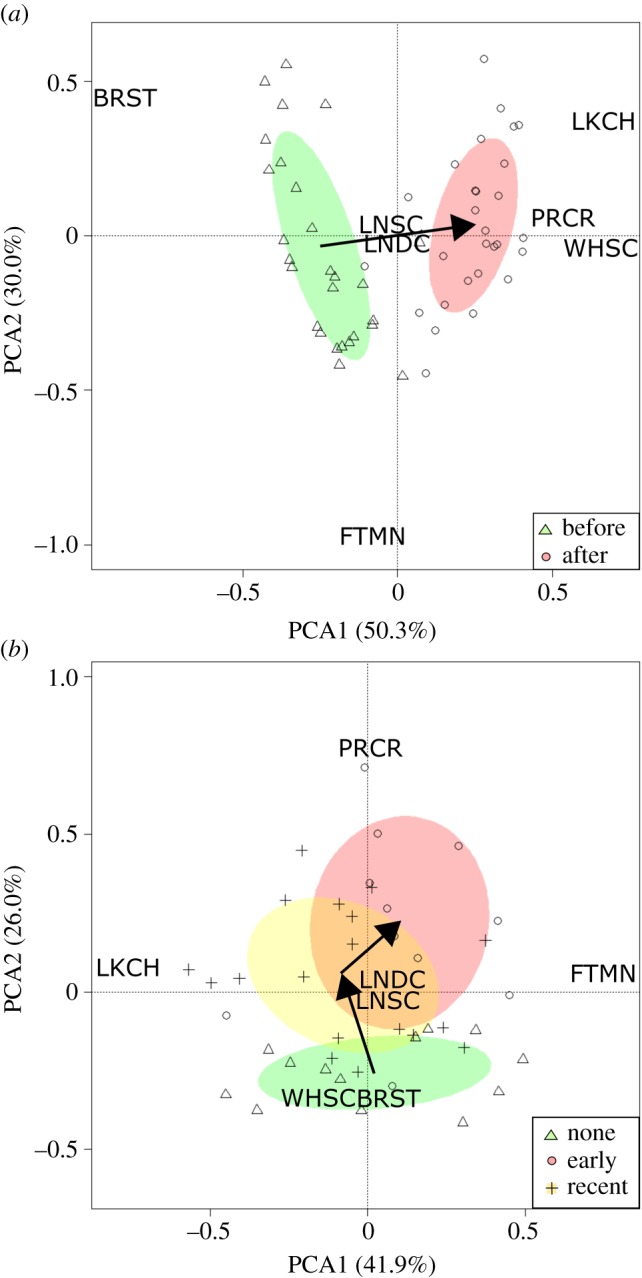
Principal components analysis of fish community composition for sites (*a*) before (2005) and after (2014) the presence of Prussian carp (*n *= 28) and (*b*) sites that have not been invaded, experienced an early invasion (4–9 years) or had a recent invasion (1–3 years) by Prussian carp (*n* = 42). Also shown are 50% confidence ellipses in (*a*) for before (green) and after (red), and in (*b*) for none (green), early (red) and recent (yellow) sites, alongside arrows that denote the temporal change in the centroid position of the ellipses. Species codes are listed in electronic supplementary material, table S2.

Comparing fish communities from before (2005) to those after (2014) Prussian carp were present, the PCA biplot explained 80.3% of variation in species composition across the first two axes of variation (figure 2*a*). We found that there were distinct differences between before and after fish communities (figure 2*a*). Of note, there was a detectable decrease in the number of brook stickleback found at sites after Prussian carp invasion (figure 2*a*). The community composition between sites before and after invasion were significantly different using a PERMANOVA (*F*_1,54 _= 49.952, *p*-value < 0.0001). Moreover, when we excluded Prussian carp from the analysis, we found significant community compositional differences between sites before and after invasion (*F*_1,54 _= 40.866, *p*-value < 0.0001). Finally, differences in the abundance of each native species before and after Prussian carp invasion demonstrated significant declines in the abundance of both brook stickleback and fathead minnows (electronic supplementary material, table S2).

Considering the time since invasion, the PCA biplot of these data explained 67.9% of variation in species composition across the first two axes of variation. The PCA biplot showed a graduated change in community composition depending on the stage of invasion (figure 2*b*). Specifically, we found significant differences between fish community composition depending on the stage of invasion (PERMANOVA *F*_2,39 _= 4.737, *p*-value = 0.0002). However, when we excluded Prussian carp from the analysis, this relationship was not significant (*F*_2,39 _= 1.4288, *p*-value = 0.22), suggesting that the abundance of Prussian carp was an important contributing factor driving differences in fish community composition. Notably, we found that the abundance of Prussian carp is significantly different when we considered the time since invasion (i.e. the longer Prussian carp have been established, the higher their abundance; electronic supplementary material, table S3). However, we also found that direct comparisons of the abundance of native species were also not significant by the time since invasion (electronic supplementary material, table S3).

### 3.2. Time since invasion impacts on native benthic invertebrates

**Figure 3.**
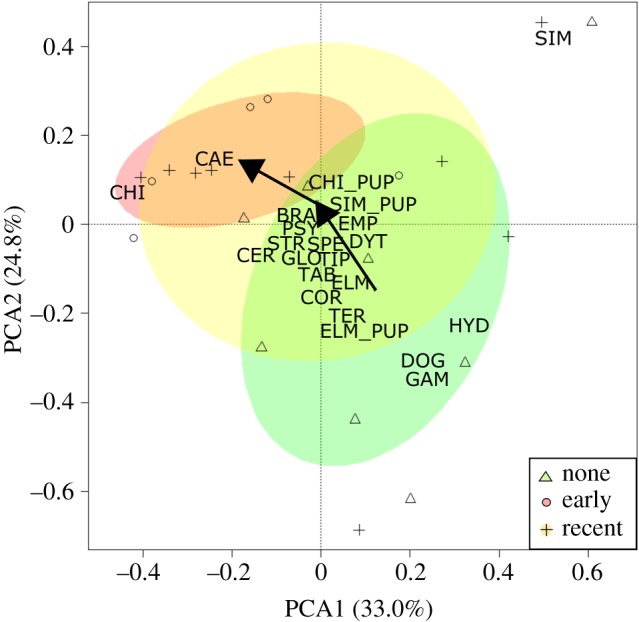
Principal components analysis of benthic invertebrate communities at sites (*n *= 22) that have not been invaded, experienced an early invasion (4–9 years) or had a recent invasion (1–3 years) by Prussian carp. Also shown are 50% confidence ellipses for none (green), early (red) and recent (yellow) sites, alongside arrows that denote the temporal change in the centroid position of the ellipses. Species codes can be found in electronic supplementary material, table S1.

We found 28 different families at a subset of sites that were sampled for benthic invertebrates (electronic supplementary material, table S1). When investigating changes in benthic invertebrates as a function of the time since invasion by Prussian carp, we found that the PCA biplot explained 57.8% of variation in community composition across the first two axes of variation (figure 3). Specifically, sites with Prussian carp present (early and recent) compared to those with Prussian carp absent showed comparatively greater differences, while differences between early and recently established sites were less distinct (figure 3). Sites with Prussian carp present appeared to have increased abundances of *Chironomidae* (larvae and pupae), *Simuliidae* (larvae and pupae) and *Caenidae* (figure 3). By contrast, sites without Prussian carp were more diverse and had higher abundances of other families of benthic invertebrates (figure 3). Using a PERMANOVA, we found that differences in the benthic invertebrate community related to the time of invasion at these sites (*n* = 22) were not significant (*F*_2,19 _= 1.699, *p*-value = 0.069). By contrast, when comparing similarities between the community composition of fish and benthic invertebrates at the site level using Procrustes analysis, we found a significant concordance between the fish and invertebrate communities (*m*_12_ = 0.758, *r* = 0.493, *p*-value = 0.047).

### 3.3. Impacts to environmental conditions and native species abundance

**Figure 4.**
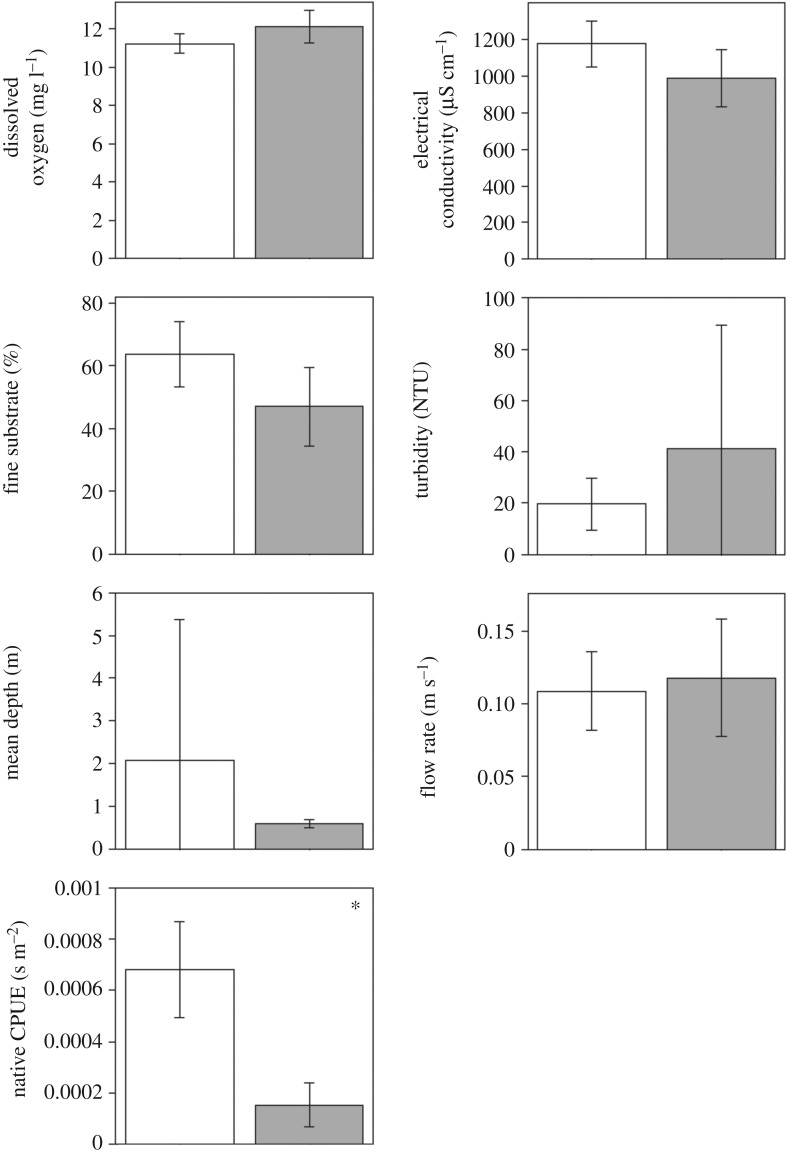
Comparison of mean differences with 95% CI in environmental characteristics at sites (*n* = 28) before (white bars) and after (grey bars) the presence of Prussian carp, corresponding to sampling in 2005 and 2014. * denotes significance at *p *< 0.05 for permuted *t*-tests.

**Figure 5.**
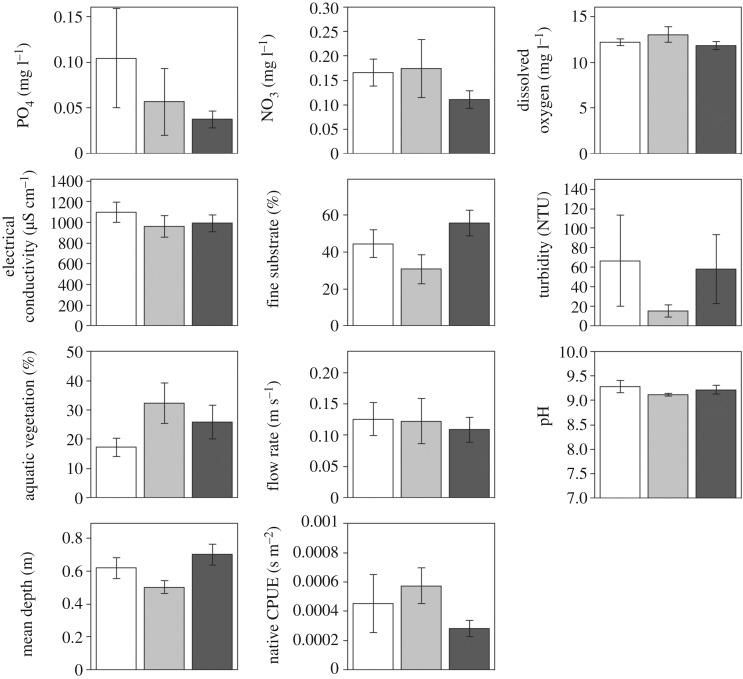
Comparison of mean differences with 95% CI in environmental characteristics measured in 2014 sampling at sites (*n* = 42) that have not been invaded (white bars), experienced an early invasion (light grey bars; 4–9 years) or had a recent invasion (dark grey bars; 1–3 years) by Prussian carp. * denotes significance at *p *< 0.05 for permuted ANOVAs.

Testing for differences in six different abiotic habitat measurements, before and after Prussian carp invasion, we found that no habitat factors were significantly different when *p*-values were adjusted for multiple comparisons (figure 4). By contrast, we found that prior to Prussian carp invasion the overall abundance of native fishes was significantly higher (figure 4; electronic supplementary material, table S2). Specifically, four times as many native fish were found at sites prior to invasion. Considering the time since invasion (none, early and recent) and testing for differences in 10 different abiotic habitat measurements, we also found no significant differences in these environmental conditions (figure 5). Moreover, we found no significant difference in the overall abundance of native fish at sites in relation to the time since invasion (figure 5; electronic supplementary material, table S3).

### 3.4. Predictors of Prussian carp abundance

Using backward selection, we determined that a GLMM with two abiotic and four biotic variables was the best fitting and most parsimonious model to predict Prussian carp abundance across our sampling sites (table 1). Specifically, Prussian carp abundance was significantly higher at sites where there was higher turbidity (table 2). Further, Prussian carp abundance was also higher in areas with more aquatic vegetation (table 2). Finally, we found that significant decreases in the abundance of fathead minnow, brook stickleback, lake chub and white sucker were significantly related to increases in Prussian carp abundance, when the time since invasion is considered (table 2).

**Table 1.** Summary of model fit for the full model and reduced model using backward selection of variables. Shown are the degrees of freedom (d.f.), log-likelihood (LL) and corrected Aikaike information criterion (AICc). See electronic supplementary material, figure S2 for species codes.

model	d.f.	LL	AICc
PRCR∼FTMN + BRST + LKCH + WHSC + LNDC + LNSC + DO + EC + fine+ turbidity + pH + depth + max_depth + flow + aquatic_veg	18	24.839	14.477
PRCR∼FTMN + BRST + LKCH + WHSC + turbidity + aquatic_veg	9	28.762	−25.250

**Table 2.** A summary of results for the best reduced generalized linear mixed model predicting the abundance of Prussian carp. Shown are the variables, associated coefficient values, *t*-value and significance. Italics denotes significant variables.

variable	coefficient	*t*-value	*p*-value
turbidity	0.047	2.432	*0*.*020*
aquatic vegetation	0.155	1.734	0.092
fathead minnow	−0.674	−3.989	*<0*.*001*
brook stickleback	−0.633	−3.881	*<0*.*001*
lake chub	−0.788	−4.274	*<0*.*001*
white sucker	−0.880	−4.463	*<0*.*001*

## 4 Discussion

In Europe, Prussian carp are considered to be a noxious invasive fish species and are regarded as more detrimental than Asian carp species, such as grass carp, which have spread across North America [14]. We present three lines of evidence that demonstrate that Prussian carp will have comparable impacts in North American freshwater ecosystems. Firstly, comparing sites before and after invasion, we demonstrated significant changes to the fish community structure, where there were direct declines in two native fish species (brook stickleback and fathead minnow). Secondly, we determined that the severity of impact on the native fish community is not significantly related to the time since invasion of Prussian carp (i.e. the longer the establishment does not equate to a greater impact) unless Prussian carp abundance is considered. This suggests that the abundance of Prussian carp may be more important to the impact on native fish communities. Notably, we found that higher abundances of Prussian carp contribute to significantly lower abundances of four out of the six native species, when partitioning the dataset by the time since invasion. Lastly, not only were native fish communities impacted, but also native benthic invertebrate communities demonstrated compositional changes alongside Prussian carp establishment. Moreover, these alterations to benthic invertebrate communities were significantly related to changes in the fish community when the time since invasion was considered. This result may be expected as wider impacts to the ecosystem by non-native species may manifest over the course of many years [52]. All of these findings are supported by similar impacts throughout their invasive range in Eurasia [14]. Altogether, given the impact of Prussian carp that we observe in North American freshwater ecosystems, this study highlights the urgency to recognize the threat posed by Prussian carp and, secondly, the need to develop conservation management schemes to lessen the impact of impending Prussian carp invasion across North America [18,53].

### 4.1. Ecosystem impacts of alterations to native biota

Prussian carp have been associated with alterations in freshwater ecosystems by altering food webs, nutrient cycling and water quality of the environment that they have invaded [15,20,21,54]. In this study, we found evidence that Prussian carp are capable of restructuring native communities of both fish and benthic invertebrates. Specifically, we documented declines of brook stickleback and fathead minnow alongside their invasion (figure 2; electronic supplementary material, table S2). Further, when Prussian carp abundance was higher, we found significantly less abundance of brook stickleback, fathead minnow, lake chub and white sucker (table 2). These findings may result from Prussian carp having similar diet and habitat preferences to brook stickleback, fathead minnow and white sucker, suggesting that Prussian carp may be introducing novel competition [18]. The negative impact on native species is surprising given that it can often take many years before community-level effects are detected [52]; however, the fact that Prussian carp do have community-level effects already demonstrates that they can be a potent invader.

Prussian carp are also known to undertake gynogenesis as a form of reproduction and populations of diploid, triploid and tetraploid individuals have been found throughout Europe [15,16]. In Europe and western Asia, declines in native cyprinids have been linked to reproductive interference from Prussian carp [55]. Minnow species (family Cyprinidae), which are the main species targeted during gynogenesis, are the most widespread and diverse family of fish in North America [56,57]. The broad distribution of native minnows makes them highly susceptible to reproductive interference and also provides ample opportunities for Prussian carp to expand its range by exploiting the sperm of native species. Additionally, native minnows may experience declines from competition for resources and habitat as Prussian carp become more abundant [18]. We found that fathead minnow and lake chub, which are both minnow species, demonstrate significant declines in abundance where Prussian carp were highly abundant (table 2). It is also possible they may be more adversely affected due to reproductive interference, because their sperm may be parasitized by the gynogenetic reproduction of Prussian carp. However, we cannot be certain whether these declines in native species abundances are related to reproductive interference or increased competition for habitat and resources [58].

Comparing the results from before–after and the time since invasion, there appears to be contrasting responses of white suckers to increases in Prussian carp abundance (figure 2). We also found that the abundance of white suckers was not significantly different when before and after establishment and the time since invasion of Prussian carp were considered (electronic supplementary material, tables S2 and S3). Thus, the observed discrepancy appears only in the before–after PCA and may be due to the number of absences of white suckers in the dataset [39,40,59]. Specifically, white suckers were detected in about half the sites before the establishment of Prussian carp compared to being observed in all of our sites after establishment. Regardless of what led to this difference, multivariate techniques such as PCA will put more weight on the absence of individuals rather than changes in their abundance, which would produce the observed discrepancy shown in our study [39]. We also note that the abundance of white suckers showed a significant decrease in response to increases in Prussian carp abundance in the GLMM, which adds more weight to the notion that white suckers may be adversely impacted by Prussian carp establishment.

Alterations to benthic invertebrate communities may be expected alongside a Prussian carp invasion, because benthic invertebrates support fish communities in the food web [42,58] and Prussian carp, in turn, consume benthic invertebrates as part of their broad diet [20,21]. Further, Prussian carp are found in higher numbers in what may be considered less suitable habitats to most native species, such as increased aquatic vegetation and turbidity (table 2), where these environmental conditions would be expected to also impact the benthic invertebrate community [60]. For instance, increased turbidity has been found to decrease overall invertebrate biomass, abundance and diversity, leading to alterations in community composition [60]. As such, we detected significant concordance between changes in the fish and benthic invertebrate community with the time since invasion, where there are increases in Chironomidae (larvae and pupae), Simuliidae (larvae and pupae) and Caenidae alongside Prussian carp invasion (figure 3). While we cannot disentangle whether changes in the benthic invertebrate community were due to the Prussian carp invasion or habitat selection, we cannot rule out that Prussian carp may be impacting benthic invertebrates, due to their diet and role as a known bioturbator (a species that reworks soils or sediments) [20,21]. Further, given this result, the time since invasion appears to be a more influential impact on the benthic invertebrate community than the fish community, as we do not detect significant differences in native fish community composition when Prussian carp are excluded from the analysis. However, these inferences should not be given much weight as the time since invasion and abundance of Prussian carp are related (electronic supplementary material, table S3). Moreover, the GLMM results suggest that when the time since invasion and Prussian carp abundance are considered in conjunction with one another, there are significant decreases in a majority of the abundances of native species when Prussian carp abundance increases. Nonetheless, Prussian carp have demonstrated that they can either impact native species by increasing in abundance or their impact may simply take more time to manifest.

### 4.2. Environmental impacts and predictors

As a bioturbator, we surprisingly find little evidence that they are altering abiotic elements of the freshwater ecosystems they have invaded [21]. This is not in agreement with many studies in Europe that report many changes in the ecosystem following invasion [14]. There are multiple reasons as to why we did not detect these impacts. Firstly, impacts to abiotic factors in the environment may be indirect and manifest at a slower rate than more direct effects on the biota within the ecosystem [61]. Secondly, we sampled lotic ecosystems and much of the work from Europe is in lentic ecosystems [15]. There are fundamental differences between these forms of freshwater ecosystems, but perhaps the most notable is the residence time of water in lentic versus lotic ecosystems. Lotic ecosystems would be continually ‘flushed’ at higher rates, whereby many localized effects of bioturbation, which would manifest as turbidity, nutrient cycling, dissolved oxygen and fine substrate, may be more difficult to detect unless impacts are system-wide. Finally, in many parts of western North America, aquatic habitat integrity is often compromised due to anthropogenic pressures from agriculture, industries and other human activities [27,62]. So, it may be difficult to detect some potential impacts of Prussian carp on abiotic conditions that would arise in more pristine systems. However, despite the study area being impacted by anthropogenic activities [62], we are still able to detect Prussian carp impacts to native species after establishment, which will have further impacts to ecosystem functioning and resilience in this system [4,5]. Thus, although the impacts of Prussian carp highlighted in this study are found to be isolated to the biota of the ecosystem, there is still the potential for much wider impacts.

Prussian carp are a species recognized for their ability to thrive in habitats less suitable for most native freshwater species (i.e. hypoxia, environmental pollution, moderate salinity, turbidity and high levels of eutrophication) [19,63,64]. Such environmental extremes were encountered during our field survey, where we found 1492 Prussian carp surviving in a small pool with only 2% dissolved oxygen recorded. Generally, Prussian carp were found in a wide range of environmental and habitat conditions in our field survey; however, Prussian carp appear to be found in greater numbers where there is higher turbidity and more aquatic vegetation, while displacing other native fish species (table 2). This is similar to observations in Eurasia, where Prussian carp are often found in slow moving and turbid environments that have aquatic vegetation [65]. In particular, dense aquatic vegetation can be an indication of eutrophication, a product of anthropogenic activities such as agriculture [66]. Prussian carp are known to flourish in eutrophic environments and tend to colonize aquatic systems as the level of eutrophication increases [23,64,67]. As aquatic habitat integrity is often compromised in many habitats in western North America [27,62], this may also facilitate Prussian carp establishment as they have the ability to exploit a potentially undesirable niche space. Although many of these fish species are relatively hardy [68], the persistence and proliferation of Prussian carp throughout North American waterways could be an additional stress on an already strained ecosystem.

### 4.3. Management implications and future directions

Ultimately, our findings are based on a correlative analysis that does not identify the specific mechanisms that are contributing to the patterns that we present in this study. Determining the impact of Prussian carp using a finer temporal resolution may provide better insight into underlying mechanisms and impacts (i.e. having a control–impact comparison through time). However, using three lines of evidence, we found consistent patterns of the impact of Prussian carp on native North American species. While these findings are informative to managers, there are still many gaps in knowledge that need to be addressed to ascertain what management prescriptions may aid in reducing their overall impact on native biodiversity. First and foremost, public education of the confirmed presence of Prussian carp and the need to prevent its human-mediated spread is fundamental to preventing further range expansion [69,70]. Secondly, the continuance of monitoring Prussian carp range expansion is needed to further understand potential barriers to its spread and the speed of the invasion [71]. Doing so, will not only highlight geographic areas affected, but allow managers to optimally allocate resources. Thirdly, elimination or subtraction studies are needed to ascertain whether active removal of Prussian carp can help reduce their impact on native biodiversity and establishment on the landscape [5]. Additionally, movement and tracking studies would help to identify the importance of human-mediated dispersal and determine if natural dispersal plays a critical role for Prussian carp range expansion, where currently to our knowledge there is very little understanding of Prussian carp movement in lotic ecosystems [72]. Finally, genetic studies would help to identify the degree to which Prussian carp are undertaking gynogenetic reproduction within their invaded range [15]. As gynogenesis parasitizes sperm from minnow species and can lead to large increases in abundance of Prussian carp [54,73], it is important to understand how much this might play a role. Addressing these knowledge gaps regarding the North American invasion would prove instrumental in preventing future range expansion.

## 5. Conclusion

Invasive species pose one of the largest threats to freshwater ecosystems [1,7]. Prussian carp have a legacy in Eurasia as a potent invader [14] and based on our results will have a similar impact in North America. Climate suitability models for Prussian carp also indicate that most of the continental United States of America provides potentially suitable habitat [53], emphasizing the need to assess the potential impact of this non-native species. Here, we demonstrate that once Prussian carp establish, they have significant negative impacts on native fish and benthic invertebrate communities. While we did not detect significant alterations to abiotic factors in lotic ecosystems (streams and rivers), this effect may be amplified in lentic ecosystems (ponds and lakes) as they have higher retention times. Given their impact on native biota, our research underlines the importance of addressing gaps in knowledge regarding this invasion and subsequently having management plans in place to reduce their overall impact. Our study suggests that a failure to do so will lead to significantly altered biodiversity and freshwater ecosystems in North America.

## Supplementary Material

Supplementary Material

## Data accessibility

Primary data can be found on Dryad (PRCR_data_July2017.xlsx; http://dx.doi.org/10.5061/dryad.ds38s) [74]. Supporting data can be found in Docherty *et al*. [18].

## Fieldwork statement

All sampling was completed with Alberta Fisheries Research Licence no. 14-1532FR. No species-at-risk were sampled in this study.
