# Supplementary material for: Native freshwater species get out of the way: Prussian carp (Carassius gibelio) impacts both fish and benthic invertebrate communities in North America
Source: R Soc Open Sci. 2017 Oct 4;4(10):170400. doi: 10.1098/rsos.170400 (PMC5666245; doi:10.1098/rsos.170400)
Supplement: Supplementary Material [file rsos170400supp1.docx]

Title: **Native freshwater species get out of the way: Prussian Carp (*Carassius gibelio*) impacts both fish and benthic invertebrate communities in North America**

Running title: **Prussian Carp impacts native biota**

Jonathan L.W. Ruppert^1^, Cassandra Docherty^1^, Kenton Neufeld^1^, Kyle Hamilton^1^, Laura MacPherson^2^, and Mark S. Poesch*^1^

^1^University of Alberta, Department of Renewable Resources, 751 General Services Building, Edmonton, AB, Canada, T6G 2H1

^2^ Alberta Environment and Parks, Fish and Wildlife Division, 6909-116 Street, Edmonton, AB, T6H 4P2

* Corresponding author: Mark S. Poesch ([poesch@ualberta.ca](mailto:poesch@ualberta.ca)); Tel.: (780) 492-4827

**Supplementary Material**

**Table S1.** Invertebrate species collected and identified to family (when possible). Shown are the identified families and species codes used in Figure 3. Families were distinguished by adults, larvae and pupae when possible. *nf* designates those that were not identified to the family level.

**Table S2.** Summary statistics of the data used in the before-after comparison. Shown are the species codes, the mean abundance of species from sites sampled before (2005) and after (2014) Prussian Carp establishment, and test statistics for permuted (*n*=10,000) *t*-tests (*df*=54; *t*-value and *p*-value). **p*-values are corrected for multiple comparisons.

| Species | Code | Before | After | *t*-value | *p*-value* |
| --- | --- | --- | --- | --- | --- |
| Prussian Carp | PRCR | 0 | 2.37E-05 | - | - |
| Brook Stickleback | BRST | 3.17E-04 | 1.41E-05 | 6.12 | 0.0006 |
| Fathead Minnow | FTMN | 3.36E-04 | 3.88E-05 | 4.25 | 0.0006 |
| Lake Chub | LKCH | 8.29E-06 | 5.68E-05 | -1.83 | 0.06 |
| Longnse Dace | LNDC | 0 | 4.04E-07 | -1.91 | 0.15 |
| Longnose Sucker | LNSC | 0 | 1.08E-06 | -1.07 | 1 |
| White Sucker | WHSC | 2.06E-05 | 3.86E-05 | -1.61 | 1 |

**Table S3.** Summary statistics of the data used in the time since establishment comparison. Shown are the species codes, the mean abundance of species from sites with none, early and recent Prussian Carp establishment, and test statistics for one-way permuted (*n*=10,000) ANOVAS (*df*=2,39; *F*-value and *p*-value). **p*-values are corrected for multiple comparisons.

| Species | Code | None | Early | Recent | *F-*value | *p*-value* |
| --- | --- | --- | --- | --- | --- | --- |
| Prussian Carp | PRCR | 0 | 0.00019 | 3.22E-05 | 5.00 | 0.0042 |
| Brook Stickleback | BRST | 6E-05 | 3.2E-05 | 3.29E-05 | 0.46 | 1 |
| Fathead Minnow | FTMN | 0.0002 | 0.00022 | 6.27E-05 | 1.15 | 1 |
| Lake Chub | LKCH | 8E-05 | 0.00015 | 9.77E-05 | 0.60 | 1 |
| Longnse Dace | LNDC | 3E-06 | 4.8E-06 | 2.36E-07 | 1.49 | 1 |
| Longnose Sucker | LNSC | 5E-07 | 6E-06 | 0 | 1.82 | 0.25 |
| White Sucker | WHSC | 0.0001 | 0.00013 | 7.21E-05 | 1.49 | 1 |

**Figure S1.** Kernel density estimates of Prussian carp range extent (dashed line) and core range (solid line) using confirmed presences from 2000-2014 (see methods and [1] for details). Shown is the kernel density and ranges for (A) 2000, (B) 2004, (C) 2006, (D) 2008, (F) 2010, (H) 2012, and (I) 2014. Crosses denote the location of sampling locations in 2014 from this study. Kernel density units are the number of presences per 0.25 square kilometers.


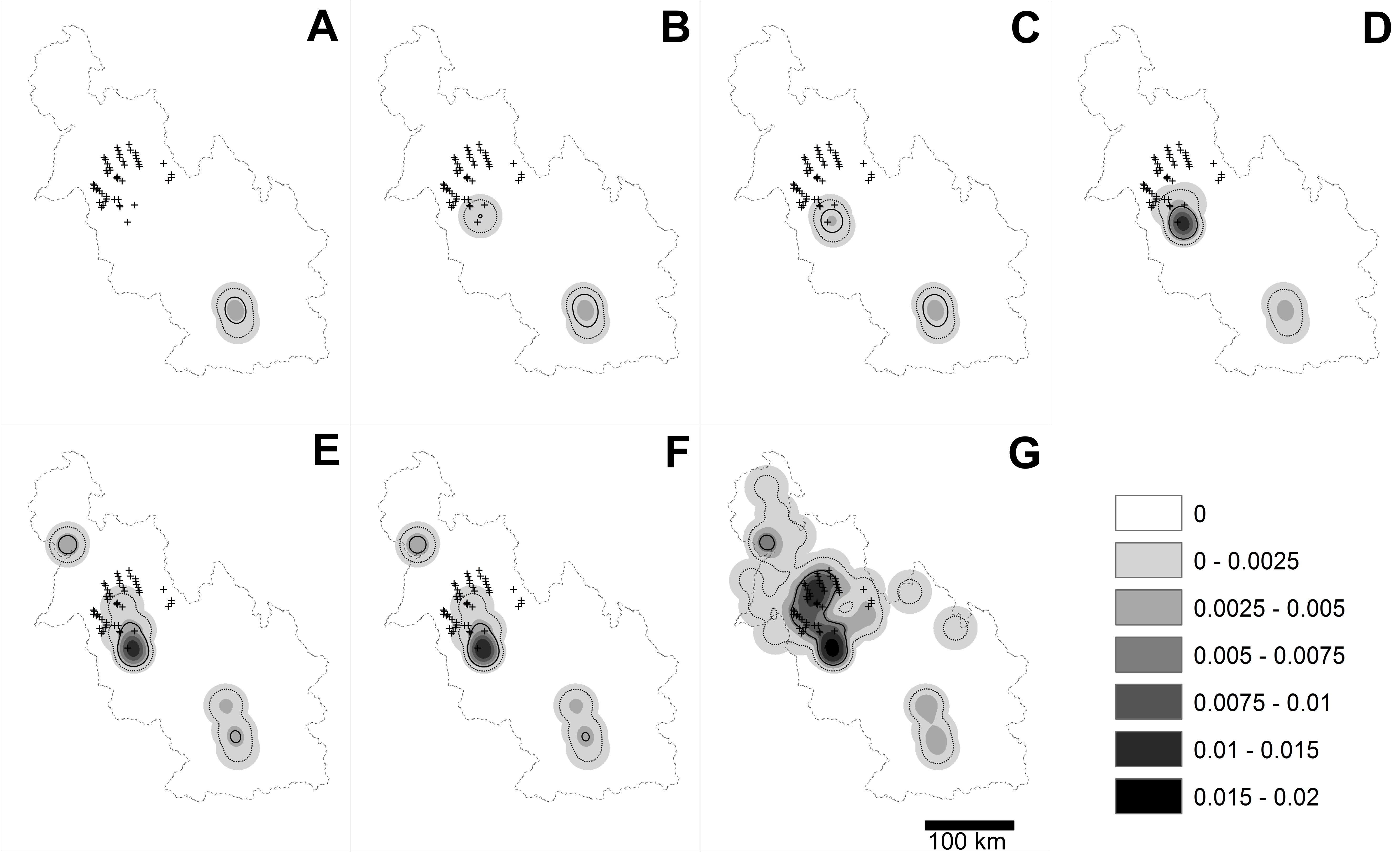


**References**

1 Docherty, C., Ruppert, J. L. W., Rudolfsen, T., Hamann, A., Poesch, M. S. In Press Assessing the spread and potential impact of Prussian Carp (Carassius gibelio Bloch, 1782) to freshwater fishes in western North America. *BioInvasions Records*.
